# Association of self-perceived income sufficiency with cognitive impairment among older adults: a population-based study in India

**DOI:** 10.1186/s12888-021-03257-4

**Published:** 2021-05-17

**Authors:** T. Muhammad, Shobhit Srivastava, T. V. Sekher

**Affiliations:** International Institute for Population Sciences, Mumbai, Maharashtra 400088 India

**Keywords:** Cognitive impairment, SES, Older adults, Regression, India

## Abstract

**Background:**

Greater cognitive performance has been shown to be associated with better mental and physical health and lower mortality. The present study contributes to the existing literature on the linkages of self-perceived income sufficiency and cognitive impairment. Study also provides additional insights on other socioeconomic and health-related variables that are associated with cognitive impairment in older ages.

**Methods:**

Data for this study is derived from the 'Building Knowledge Base on Population Ageing in India'. The final sample size for the analysis after removing missing cases was 9176 older adults. Descriptive along with bivariate analyses were presented to show the plausible associations of cognitive impairment with potential risk factors using the chi-square test. Also, binary logistic regression analysis was performed to provide the relationship between cognitive impairment and risk factors. The software used was STATA 14.

**Results:**

About 43% of older adults reported that they had no source of income and 7.2% had income but not sufficient to fulfil their basic needs. Older adults with income but partially sufficient to fulfil their basic needs had 39% significantly higher likelihood to suffer from cognitive impairment than older adults who had sufficient income [OR: 1.39; OR: 1.21–1.59]. Likelihood of cognitive impairment was low among older adults with asset ownership than older adults with no asset ownership [OR: 0.83; CI: 0.72–0.95]. Again, older adults who work by compulsion (73.3%) or felt mental or physical stress due to work (57.6%) had highest percentage of cognitive impairment. Moreover, older adults with poor self-rated health, low instrumental activities of daily living, low activities of daily living, low subjective well-being and low psychological health were at increased risk for cognitive impairment.

**Conclusion:**

The study highlights the pressing need for care and support and especially financial incentives in the old age to preserve cognitive health. Further, while planning geriatric health care for older adults in India, priority must be given to financially backward, with no asset ownership, with poor health status, older-older, widowed, and illiterate older individuals, as they are more vulnerable to cognitive impairment.

## Background

Aging population is posited as a social challenge at global level. It reflects in older ages at which mental functioning becomes significantly impaired [1]. Cognitive impairment, including dementia as one outcome of decline in cognitive ability increases considerably with the rapidly growing population of older adults [2]. Worldwide, almost 80% of the general public are concerned about developing dementia at some point of time and 1 in 4 people think that they can do nothing to prevent dementia [3]. Further, evidence suggests an aggregate effect of socio-economic risk factors on cognitive impairment among older adults [4, 5].

Persons with higher cumulative socio-economic status (SES) demonstrated an advantage in cognitive functioning [6]. Growing body of literature suggests that the older adults with higher accumulation of wealth may be able to more easily translate it into better circumstances or less stressful living conditions, further contributing to better cognitive health in later life [5, 7–9]. Studies in developing countries have also demonstrated that social networks and familial support are major factors that provide older adults mental reserves and enhance their wellbeing especially those from poor resource settings [10–12]. On the other hand, people with low income, low education, lack of support networks or lack of access to appropriate health or social services are all at a greater risk for poor health conditions that are, in turn, risk factors for declining cognitive ability [13]. However, studies reported improvements in mental well-being for older people after the introduction of an income supplemental program [14, 15], indicating that individual income status has a major contribution to late life wellbeing. Similarly, recent research has revealed that self-perceived income sufficiency assessment is a useful tool both theoretically and practically, in an underserved population where participants may be more reluctant to report their income levels than their perceived income status [16].

Furthermore, a major contributing factor may include poor literacy resulting in an inability to benefit from resources on strategies to early prevention of cognitive impairment [17–19]. In multiple studies that measured late-life cognitive impairment by a test of processing speed, it was found that educational attainment and current poverty index were associated with cognition [5, 20, 21]. Furthermore, each SES indicator shows different financial resources, social prestige and diverse skills of an individual [4]. While the spousal loss can cause emotional distress accompanied by bereavement, widowhood status accelerates cognitive impairment over time among widowed older adults [22, 23]. Studies also found that difficulty in activities of daily living, poor self-reported health, status of being bedridden and depressive symptoms are significantly associated with cognitive impairment [24, 25]. Further, multi-morbidity and sematic comorbidities were also associated with faster declines in geriatric cognition [26].

Greater socioeconomic and gender disparities in health among older individuals in India have been well investigated [27–29]. Much studies in other countries have established that poor financial status was associated with worse cognitive function due to limited resources and health-related deprivations [9, 30, 31]. Previous studies on cognitive functioning among older adults in India focussed on gender, work status and regional variations [32–34]. However, the association of several social, economic and health-related factors with cognitive impairment that may help identify the intervention strategies that are likely to result in better cognitive health and a successful ageing is poorly understood in Indian context.

Since greater cognitive performance has been shown to be associated with better mental and physical health and lower mortality [35, 36]. The present study contributes to the existing literature on the linkages of self-perceived income sufficiency and cognitive impairment. Study also provides additional insights on socioeconomic and health status that is associated with cognitive impairment in older ages by assessing a population based survey in India. We hypothesize that:

H_1_: Self-perceived income sufficiency of older adults is positively associated with cognitive functioning, independent of other socio-demographic and health-related variables.

H_2_: Older adults’ working status is positively associated with cognitive impairment.

H_3_: Education, asset ownership and household economic status would each be associated with cognitive functioning among older adults, such that higher levels of each would predict better cognitive status.

## Data and methods

### Data

Data for this study is derived from the BKPAI (Building Knowledge Base on Population Ageing in India) which was carried out in India. A primary survey was carried out in seven states of India (Himachal Pradesh, Punjab, West Bengal, Odisha, Maharashtra, Kerala and Tamil Nadu), that covered a total of 9852 elders from 8329 elderly households in rural and urban areas. As these states have a higher percentage of the 60+ population compared to the national average and these states also represent all regions of the country. The individual’s questionnaire was used which covers on the socio-demographic profile, work history, and benefit, income, and assets, living arrangement, social activities, the health status of elderly & social security related question [37]. The BKPAI sample design entails a two-stage probability sampling. Where first villages were classified into different strata on the basis of population size, and the number of primary sampling units (PSUs) to be selected was determined in proportion to the population size of each stratum. Using probability proportional to population size (PPS) technique, the PSUs have been chosen, and within each selected PSU, elderly households were selected through systematic sampling. A similar procedure was applied in drawing samples from urban areas [37]. The final sample size for the analysis after removing missing cases was 9176 older adults.

### Variable description

#### Outcome variable

The outcome variable was binary and was assessed though verbal recall strategy in the questionnaire [37, 38]. To measure cognitive impairment, a scale of 0 to 10 was prepared representing higher the score lower the cognitive impairment [10]. Five or more words were recoded as 0 “low” representing lower cognitive impairment and score of four or less was recoded as 1 “high” representing higher cognitive impairment [1]. Coginitve impairment represents poor coginitve ability among older adults. This survey has used immediate recall of words to assess the degree of cognitive impairment among older adults [1]. Immediate recall has worked as a reliable test to measure cognitive ability among elderly in different studies [39–41]. The words used for testing cognitive impairment were Bus, House, Chair, Banana, Sun, Bird, Cat, Saree, Rice, and Monkey which are commonly used in Indian scenario [41].

#### Explanatory variable

Self-perceived income sufficiency was recoded as (no income, has income and fully sufficient, has income and partially sufficient and has income and not sufficient), working status was recoded as (never worked, currently working and retired), educational status was recoded as (no education, below five years, 6–10 years and 11+ years), marital status was recoded as (not in union and currently in union), asset ownership was asked regarding home ownership, land ownership, jewellery ownership and other monetary savings and was recoded as (no and yes), age was recoded as (60–69 years, 70–79 years and 80+ years). Co-residing with children was recoded as (no and yes).

Self-rated health (SRH) was having a scale of 1 to 5 “poor to excellent” and was categorized as 0 “good” (representing good, very good and excellent) and 1 “poor” (representing poor or fair) [42]. Ability to do activities of daily living (ADL) was having a scale of 0 to 6 where in it represents higher the score higher the independence. A score of was categorized as 0 “high” which represents full independence and 5 and less was categorized as 1 “low” which represents not fully independent to do activities of daily living (Cronbach Alpha: 0.93) [43]. Ability to do instrumental activities of daily living (IADL) was having a scale of 0 to 8 representing higher the score higher the independence. A score of 6+ was categorized as 0 “high” representing high IADL and score of 5 and less was recoded as 1 “low” representing low IADL [40].

The 12-item version of the General Health Questionnaire (GHQ-12) was used as a measure of low psychological health. Developed by Goldberg in the 1970s, this measure has been extensively used to measure the psychological or mental health status in different settings and different cultures [44]. Psychological health was having a scale of 0 to 12 on the basis of experiencing stressful symptoms and was recoded as 0 “high” (representing 6+ scores) and 1 “low” (representing score 5 and less) [45, 46]. The low psychological health represents lower levels of psychological health or psychological distress among older adults (Cronbach alpha: 0.90). The 9-item subjective well-being questionnaire was used to measure low subjective well-being. Subjective wellbeing was having a scale of 0 to 9 and was categorized as 0 “high” experiencing better experience (representing 6+ scores) and 1 “low” experiencing negative experience (representing score 5 and less) [47]. Twelve questions on psychological health and nine questions on subjective well-being were asked to assess the outcome. All the questions were asked on Likert scales and were recoded and used accordingly as per literature [10, 40]. The low subjective well-being represents lower levels of subjective well-being among older adults (Cronbach alpha: 0.93).

Wealth index was categorized in five quintile i.e. poorest, poorer, middle, richer and richest [48], religion was recoded as Hindu, Muslim, Sikhs and others, caste was available as Scheduled Caste, Scheduled Tribe, Other Backward Class and Other [48] and place of residence was available as rural and urban. Data was collected in seven states of India to make it representable i.e., Himachal Pradesh, Punjab, West Bengal, Odisha, Maharashtra, Kerala and Tamil Nadu.

### Statistical analysis

Descriptive analysis along with bivariate analysis was used to find the plausible association between cognitive impairment with exposure and potential risk factors using the chi-square test [49]. Apart from binary logistic regression analysis [50] was used to provide the relationship between cognitive impairment and other risk factors. The software used was STATA 14. The significance level was set to be 5% (*p* < 0.05. R-Squared is the proportion of variance in the dependent variable (cognitive impairment) which can be predicted from the independent variables. When analysing data with a logistic regression, an equivalent statistic to R-squared does not exist. The model estimates from a logistic regression are maximum likelihood estimates arrived at through an iterative process. They are not calculated to minimize variance, so the OLS approach to goodness-of-fit does not apply. However, to evaluate the goodness-of-fit of logistic models, several pseudo R-squared have been developed. A pseudo R-squared only has meaning when compared to another pseudo R-squared of the same type, on the same data, predicting the same outcome. In this situation, the higher pseudo R-squared indicates which model better predicts the outcome [51, 52]. Additionally, variance inflation factor was computed to check the multicollinearity among the variables used [53, 54]. It was found that there was no evidence of multicollinearity among the variables used in this study. Moreover, svyset command [55] in STATA 14 [56] was used to control for complex survey design. Individual level weights which were available in the dataset were used during the analyses.

## Results

Table 1 represents the socio-economic and demographic profile of older adults in India. Responding to the question on self-perceived income sufficiency, about 43% of older adults reported that they had no source of income. Nearly 67% of older adults did not work in the last one year period. Almost half of the older adults had no education at the time of survey. Nearly 40% of older adults were not in a marital union. About 18% of older adults had no asset ownership. One in tenth of the older adults were from the age group 80 years and above. More than 50% of older adults were women and slightly less than 50% were men. About 29% of older adults were not co-residing with their children. More than half of the older adults in India reported that they had poor SRH; about 57% reported that they had low IADL and about 7% had low ADL. Nearly 27% and 24% had low subjective well-being and low psychological health. About 24% of older adults belong to poorest wealth status and 15% belong to richest wealth status. Nearly, 80% of the population belong to Hindu religion and 21% of the study population belong to Scheduled Caste category. About 26% of the study population belong to urban areas.

**Table 1:** Socio-economic and demographic profile of older adults in India

Variables	Sample	Percentage
**Self-perceived income sufficiency**		
Has income and fully sufficient	2156	23.5
Has income and partially sufficient	2410	26.3
Has income and not sufficient	661	7.2
No income	3949	43.0
**Working status (last one year)**		
Never worked	6174	67.3
Currently working	2208	24.1
Retired	794	8.7
**Educational status**		
No education	4654	50.7
Below 5 years	1890	20.6
6 to 10 years	2072	22.6
11+ years	559	6.1
**Marital status**		
Not in union	3632	39.6
Currently in union	5544	60.4
**Asset ownership**		
No	1610	17.6
Yes	7566	82.5
**Age group (in years)**		
60–69	5667	61.8
70–79	2525	27.5
80+	984	10.7
**Sex**		
Men	4339	47.3
Women	4837	52.7
**Co-residing with children**		
No	2701	29.4
Yes	6475	70.6
**Self-rated health**		
Good	4096	44.6
Poor	5080	55.4
**IADL**		
High	3995	43.5
Low	5181	56.5
**ADL**		
High	8498	92.6
Low	678	7.4
**Subjective well-being**		
High	6720	73.2
Low	2456	26.8
**Psychological health**		
High	7024	76.6
Low	2152	23.5
**Wealth status**		
Poorest	2170	23.7
Poorer	2024	22.1
Middle	1903	20.7
Richer	1708	18.6
Richest	1370	14.9
**Religion**		
Hindu	7299	79.6
Muslims	644	7.0
Sikh	847	9.2
Others	386	4.2
**Caste**		
Scheduled Caste	1897	20.7
Scheduled Tribe	515	5.6
Other Backward Class	3353	36.5
Others	3411	37.2
**Place of residence**		
Rural	6783	73.9
Urban	2393	26.1
**State**		
Himachal Pradesh	1456	15.9
Punjab	1240	13.5
West Bengal	1127	12.3
Orissa	1453	15.8
Maharashtra	1230	13.4
Kerala	1340	14.6
Tamil Nadu	1330	14.5
**Total**	9176	100.0

*IADL* Instrumental activities of daily living, *ADL* Activities of daily living

Table 2 presents percentage of older adults with cognitive impairment by background characteristics among older adults in India. Older adults who had income but not sufficient to fulfil their basic needs had highest percentage of cognitive impairment (71.1%). Older adults who never worked had highest percentage of cognitive impairment (66.0%). Older adults with no education (70.6%) had highest percentage of cognitive impairment. Being not in a marital union (68.9%) was a significant risk factor for cognitive impairment. About 71.6% of older adults with no asset ownership had cognitive impairment in comparison to 57.5 with asset ownership. Oldest old (78.5%), older women (66.3%) and older adults co-residing with children (60.9%) had higher percentage of cognitive impairment. Older adults with poor SRH (69%), low IADL (68.0%), low ADL (87.7%), low subjective well-being (74.6%) and low psychological health (76.5%) had higher percentage of cognitive impairment. Being from a poorest wealth status (71.2%) possesses the extreme risk of high percentage of cognitive impairment among older adults. Older adults from rural background (63.0%) had higher percentage of cognitive impairment.

**Table 2:** Percentage of older adults with cognitive impairment by background characteristics among older adults in India

Variables	Percentage	p < 0.05
**Self-perceived income sufficiency**		*
Has income and fully sufficient	43.5	
Has income and partially sufficient	65.6	
Has income and not sufficient	71.1	
No income	63.8	
**Working status (last one year)**		*
Never worked	66.0	
Currently working	53.0	
Retired	32.8	
**Educational status**		*
No education	70.6	
Below 5 years	63.6	
6 to 10 years	40.8	
11+ years	31.0	
**Marital status**		*
Not in union	68.9	
Currently in union	54.2	
**Asset ownership**		*
No	71.6	
Yes	57.5	
**Age group (in years)**		*
60–69	53.1	
70–79	68.2	
80+	78.5	
**Sex**		*
Men	53.0	
Women	66.3	
**Co-residing with children**		*
No	57.8	
Yes	60.9	
**Self-rated health**		*
Good	48.9	
Poor	69.0	
**IADL**		*
High	49.6	
Low	68.0	
**ADL**		*
High	58.0	
Low	84.7	
**Subjective well-being**		*
High	54.7	
Low	74.6	
**Psychological health**		*
High	55.0	
Low	76.5	
**Wealth status**		*
Poorest	71.2	
Poorer	65.1	
Middle	60.1	
Richer	48.7	
Richest	48.7	
**Religion**		*
Hindu	59.6	
Muslims	67.1	
Sikh	56.1	
Others	64.0	
**Caste**		*
Scheduled Caste	66.2	
Scheduled Tribe	71.4	
Other Backward Class	56.5	
Others	58.3	
**Place of residence**		*
Rural	63.0	
Urban	51.6	
**State**		*
Himachal Pradesh	54.2	
Punjab	54.9	
West Bengal	81.9	
Orissa	69.3	
Maharashtra	55.0	
Kerala	66.3	
Tamil Nadu	40.7	
**Total**	60.0	

*if *p* < 0.05; IADL: Instrumental activities of daily living; ADL: Activities of daily living

**Fig. 1:**
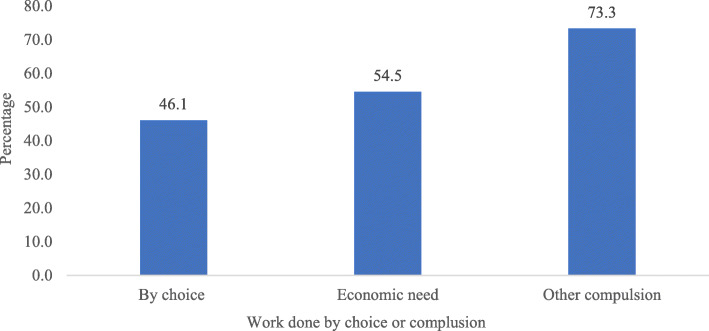
Percentage of cognitive impairment among older adults by their working status as by choice, economic need or compulsion

Figure 1 represents percentage of cognitive impairment among older adults by their working status as by choice or compulsion. Older adults who work by other compulsions (73.3%) had highest percentage of cognitive impairment.

**Fig. 2:**
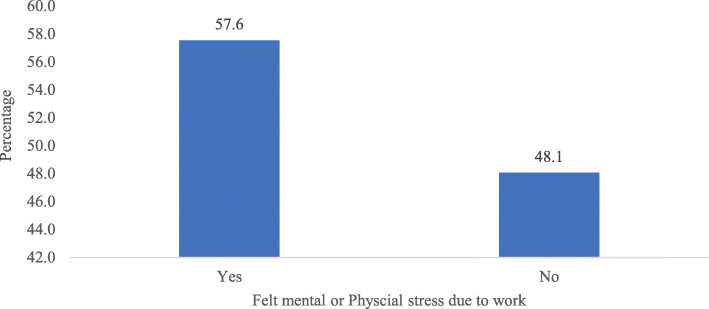
Percentage of cognitive impairment among older adults by their mental or physical stress due to work

Figure 2 reveals percentage of cognitive impairment among older adults by their mental or physical stress due to work. Older adults who felt mental or physical stress (57.6%) had highest percentage of cognitive impairment.

Table 3 represents logistic regression estimates for cognitive impairment by background characteristics among older adults in India. The table consists of four models. Model-1 holds individual level factors including demographic factors. Model-2 holds health factors which are at individual level but focus on health issues. Model-3 holds household level factors and model-4 is the full effect model. The model fit is explained using pseudo R square and Negelkerke R-square which explain the variance in the model. R-squared measures the strength of the relationship between the model and the dependent variable on a convenient 0–100% scale. Model-1 followed by model-3 and model-2 explains the variation for cognitive impairment. However, model-4 had the highest R square values as it is the full effect model. This signifies that individual level factors followed by household and health factors explain the variation in cognitive impairment among older adults in India.

**Table 3:** Logistic regression estimates for cognitive impairment by background characteristics among older adults in India

Variables	Model-1	Model-2	Model-3	Model-4
	OR (95% CI)	OR (95% CI)	OR (95% CI)	OR (95% CI)
**Self-perceived income sufficiency**				
Has income and fully sufficient	Ref.			Ref.
Has income and partially sufficient	1.92*(1.69,2.19)			1.39*(1.21,1.59)
Has income and not sufficient	2.37*(1.92,2.92)			1.34*(1.07,1.68)
No income	1.50*(1.30,1.72)			1.25*(1.07,1.45)
**Working status (last one year)**				
Never worked	Ref.			Ref.
Currently working	0.84*(0.74,0.97)			0.88 (0.76,1.02)
Retired	0.64*(0.53,0.77)			0.77*(0.63,0.94)
**Educational status**				
No education	Ref.			Ref.
Below 5 years	0.74*(0.65,0.83)			0.69*(0.6,0.79)
6 to 10 years	0.40*(0.36,0.45)			0.45*(0.39,0.52)
11+ years	0.27*(0.22,0.32)			0.28*(0.22,0.35)
**Marital status**				
Not in union	Ref.			Ref.
Currently in union	0.80*(0.72,0.89)			0.84*(0.75,0.95)
**Asset ownership**				
No	Ref.			Ref.
Yes	0.71*(0.63,0.81)			0.83*(0.72,0.95)
**Age group (in years)**				
60–69	Ref.			Ref.
70–79	1.65*(1.48,1.84)			1.48*(1.32,1.66)
80+	2.38*(2,20.83)			1.92*(1.59,2.32)
**Sex**				
Men	Ref.			Ref.
Women	0.91 (0.81,1.03)			1.04 (0.92,1.18)
**Co-residing with children**				
No	Ref.			Ref.
Yes	0.96 (0.87,1.07)			0.95 (0.85,1.07)
**Self-rated health**				
Good		Ref.		Ref.
Poor		1.75*(1.60,1.92)		1.28*(1.15,1.42)
**IADL**				
High		Ref.		Ref.
Low		1.72*(1.58,1.88)		1.26*(1.13,1.40)
**ADL**				
High		Ref.		Ref.
Low		2.19*(1.76,2.72)		1.91*(1.51,2.42)
**Subjective well-being**				
High		Ref.		Ref.
Low		1.57*(1.40,1.77)		1.13*(1.04,1.29)
**Psychological health**				
High		Ref.		Ref.
Low		1.56*(1.37,1.77)		1.58*(1.37,1.82)
**Wealth status**				
Poorest			Ref.	Ref.
Poorer			0.88 (0.76,1.02)	1.08 (0.92,1.27)
Middle			0.64*(0.55,0.75)	0.93 (0.78,1.11)
Richer			0.43*(0.37,0.51)	0.71*(0.59,0.86)
Richest			0.32*(0.27,0.38)	0.69*(0.56,0.85)
**Religion**				
Hindu			Ref.	Ref.
Muslims			1.21 (0.99,1.47)	0.90 (0.73,1.11)
Sikh			1.04 (0.84,1.30)	0.93 (0.73,1.17)
Others			1.27*(1.01,1.59)	1.30*(1.02,1.65)
**Caste**				
Scheduled Caste			Ref.	Ref.
Scheduled Tribe			0.85 (0.68,1.08)	1.01 (0.79,1.29)
Other Backward Class			0.89 (0.77,1.02)	0.98 (0.84,1.14)
Others			0.82*(0.72,0.93)	0.96 (0.84,1.11)
**Place of residence**				
Rural			Ref.	Ref.
Urban			0.80*(0.72,0.88)	0.90 (0.81,1.01)
**State**				
Himachal Pradesh			Ref.	Ref.
Punjab			1.33*(1.09,1.63)	1.16 (0.93,1.44)
West Bengal			4.14*(3.42,5.02)	4.40*(3.55,5.45)
Orissa			1.64*(1.39,1.95)	1.65*(1.37,1.99)
Maharashtra			1.04 (0.88,1.23)	1.15 (0.96,1.38)
Kerala			2.09*(1.75,2.5)	2.35*(1.93,2.87)
Tamil Nadu			0.57*(0.48,0.69)	0.59*(0.48,0.72)
**Pseudo R-Square**	0.11	0.07	0.08	0.17
**Negelkerke R-Square**	0.18	0.13	0.15	0.28

*OR* Odds Ratio, *CI* Confidence Interval; *if *p* < 0.05; *IADL* Instrumental activities of daily living, *ADL* Activities of daily living; Ref: Reference

Older adults with income but partially sufficient to fulfil their basic needs had 39% significantly higher likelihood to suffer from cognitive impairment than older adults who had income that was fully sufficient [OR: 1.39; OR: 1.21–1.59]. Older adults who were retired had 23% significantly lower likelihood to suffer from cognitive impairment than older adults who did not work in the last one year period [OR: 0.77; CI: 0.63–0.94]. Older adults with 11 and more years of education had 72% significantly lower likelihood to suffer from cognitive impairment than older adults who were uneducated [OR: 0.28; CI: 0.22–0.35]. Older adults who were currently in a marital union had 16% significantly lower likelihood to suffer from cognitive impairment than older adults not in a union.

Likelihood of cognitive impairment was low among older adults with asset ownership than older adults with no asset ownership [OR: 0.83; CI: 0.72–0.95]. Being in oldest old age group, the odds of cognitive impairment was high among such population [OR: 1.92; CI: 1.59–2.32]. Older adults with poor SRH [OR: 1.28; CI: 1.15–1.42], low IADL [OR: 1.26; CI: 1.13–1.40], low ADL [OR: 1.91; CI: 1.51–2.42], low subjective well-being [OR: 1.13; CI: 1.04–1.29] and low psychological health [OR: 1.58; CI: 1.37–1.82] had higher likelihood to suffer from cognitive impairment than their counterparts. Older adults from richest wealth status had significantly lower likelihood to suffer from cognitive impairment than older adults from poorest wealth status [OR: 0.69; CI: 0.56–0.85]. Older adults from West Bengal [OR: 4.40; CI: 3.55–5.45], Kerala [OR: 2.35; CI:1.93–2.87] and Orissa [OR: 1.65; CI: 1.37–1.99] had significantly higher odds to suffer from cognitive impairment than older adults from Himachal Pradesh.

## Discussion

The study used a large cross-sectional data to investigate the association of major socio-economic and health-related variables with cognitive impairment among older adults. Our study made a few important findings, which provide a comprehensive understanding of the impact of socio-economic deprivations and worse health status on cognitive impairment among older people. Results of the study revealed that older individual’s perceived income status that is sufficient and meets their basic needs was associated with better cognitive functioning. Our finding is supported by a recent study that found that self-perceived income status is positively associated with life satisfaction, happiness, and overall mental wellbeing [57]. Many previous studies have found that poor financial status was associated with worse cognitive functioning [2, 9, 31, 58]. A longitudinal study on health benefits associated with the additional income shows that relative to the control site, there was a statistically significant improvement in memory and overall health of older adults who are provided with an additional income [59]. Participants in the same study used their extra income to go to the doctor, buy their medications, and alleviate their hunger and with basic health improvements, their cognitive abilities started to improve.

Another key SES indicator that is analysed in this population-based study is working status of older adults. Results show that older adults who had never worked for last one year had the highest odds for impaired cognition compared to those who are retired or currently working after adjusting for socio-economic and health factors. Moreover, another interesting picture appears with the higher chances of cognitive impairment among currently working older adults than those who are retired. Since a large proportion of older adults in present study continue to engage in work by compulsion or to overcome financial distress and were associated with mental or physical stress, the results do not support the notion that current working status is associated with better cognitive outcome [60]. The finding is also contrary to studies in developed countries that demonstrated that working in an occupation characterized by higher levels of mental demands was associated with better cognitive functioning before retirement, and a slower rate of cognitive impairment after retirement [61, 62]. However, in consistence with our finding, recent studies in developing countries suggest that retirement also provides a fair opportunity to engage in long-pending social activities, also known as the ‘honeymoon effect’ and reduce negative feelings so as to optimize cognitive functioning [63–65]. Other research suggests that retirement and involvement in voluntary activities, meeting with relatives, and participation in other social activities results in cognitive preservation [66]. Although many studies including ours establish an association of working status with cognitive functioning, inconsistencies remain in most studies on the causality. Thus, longitudinal studies are further warranted in different cultural settings.

Further, current income status may be a poor indicator of financial resources because income may drop with retirement even if wealth is at its life-time peak [67]. Particularly for older adults, income is a less complete assessment of economic status than wealth is, because it does not reflect the value of accumulated assets such as home and other property [68]. after controlling the socio-demographic and health factors, it was found that having no asset ownership had a statistically significant association with cognitive impairment. The finding is supported by a study that showed better cognitive performance of older adults who had accumulated substantial wealth as asset just as they approached the point in their life cycle when they rely on their wealth to protect them against the increasing risk of health events [7]. However, a voluminous literature suggests that education has the strongest association with cognitive functioning, followed by current material circumstances [1, 4, 69]. Concordantly, our study also found a statistically significant positive association between higher-educated older adults and better cognitive functioning compared to those without formal education. Although the education–cognition relationship may in part reflect an SES gradient, the association is more likely due to the process and consequences of education itself [70]. education benefits cognitive health in later life primarily by providing cognitive reserve, which in turn provides resilience to age-related neuropathology [69]. A recent study found that individuals having low levels of education and those belonging to poorer wealth quintiles experience a stressful impact on cognition in old age [65].

Marital status in the present study was significantly associated with cognitive impairment. It is possible that sharing one’s life with a partner results in stimulating brain activities and as a result, married persons could have lower speed of cognitive decline [71]. Further, because of the stigma of dependency and care burden, many older parents are reluctant to ask for financial assistance or care from their adult children [39, 72] indicating that co-residence with children may not be a protective factor of cognitive impairment in old age. Consistently, we observed no statistically significant association of co-residence with cognitive impairment in present study. In agreement with earlier studies, higher cognitive impairment in present study was observed in older adults with low subjective health, low psychological health, poor SRH and deterioration in ADL and IADL. Further, subjective losses of daily functioning have been described as features of mild cognitive impairment and the significant association is well documented in many previous studies [73–76]. Our findings consistent with previous studies, suggest that in a low socioeconomic setting with no awareness or preparedness for mental health problems, Western models of care arrangements would be inappropriate, therefore, family-based care settings could be a way forward [77, 78].

The present study however had certain limitations. Since the results of this study are based on cross-sectional data, study limits our ability to establish causality. Further, the data was collected from seven states of India which represents six regions of India. Therefore, even being a nationally representative study, one has to be cautious while generalizing the results. However, study has its own strengths too. The study includes large sample size with a national representation and the data is rich with information on older adults’ self-perceived income status and ageing-related issues in Indian scenario.

## Conclusion

Our findings suggest that poor economic status is independently associated with cognitive impairment in Indian adults over age 60 years. Older adults with lower levels of any of the health indicators were at increased risk for cognitive impairment whereas, higher levels of education and household wealth status were major protective factors of cognitive impairment in old age. Thus, the study highlights the pressing need for care and support and especially financial incentives in the old age to preserve cognitive health. While planning geriatric health care for older adults, priority must be given to financially backward, with no asset ownership, with poor health status, older-older, widowed, and illiterate older individuals, as they are more vulnerable to cognitive impairment.

To reduce cognitive impairment in later life, policy makers should pay more attention to reducing socio-economic deprivation by implementing programs and policies based on each component of SES separately. Policies increasing social capital by educating, increasing access to health care, reducing economic inequality, and promoting positive health- related behaviours may enhance the cognitive health of low-SES individuals. Moreover, establishing an early diagnosis may enable older adults and their family members prepare for the future in an appropriate way. Also, further investigation is required to examine the sociocultural and regional differences in the association of SES and cognitive ageing that can help discern factors and inform the development of preventive strategies.

## Data Availability

The study utilises secondary source of data which is freely available in public domain through http://www.isec.ac.in/

## Abbreviations

SRH: Self-rated health
ADL: Activities of daily living
IADL: Instrumental activities of daily living
OR: Odds ratio
CI: Confidence Interval
BKPAI: Building a Knowledge Base on Population Aging in India
